# Molecular Characterization and Virus-Induced Gene Silencing of a Collagen Gene, *Me-col-1*, in Root-Knot Nematode *Meloidogyne enterolobii*

**DOI:** 10.3390/life12122103

**Published:** 2022-12-14

**Authors:** Ji Pei, Tuizi Feng, Haibo Long, Yuan Chen, Yueling Pei, Yanfang Sun

**Affiliations:** 1 College of Plant Protection/Key Laboratory of Green Prevention and Control of Tropical Plant Diseases and Pests, Ministry of Education, Hainan University, Haikou 570228, China; 2 Key Laboratory of Integrated Pest Management on Tropical Crops, Ministry of Agriculture and Rural Affairs, Institute of Environment and Plant Protection, Chinese Academy of Tropical Agricultural Sciences, Haikou 571101, China

**Keywords:** *Meloidogyne enterolobii*, root-knot nematode, collagens, VIGS, RNAi

## Abstract

*Meloidogyne enterolobii*, a highly pathogenic root-knot nematode species, causes serious damage to agricultural production worldwide. Collagen is an important part of the nematode epidermis, which is crucial for nematode shape maintenance, motility, and reproduction. In this study, we report that a novel collagen gene, *Me-col-1,* from the highly pathogenic root-knot nematode species *Meloidogyne enterolobi* was required for the egg formation of this pathogen. *Me-col-1* encodes a protein with the size of 35 kDa, which is closely related to collagen found in other nematodes. Real-time PCR assays showed that the expression of *Me-col-1* was highest in eggs and lowest in pre-parasitic second-stage juveniles (preJ2). Interestingly, knockdown of *Me-col-1* did not compromise the survival rate of preJ2 but significantly reduced the egg production and consequentially caused 35.79% lower multiplication rate (Pf/Pi) compared with control. Our study provides valuable information for better understanding the function of collagen genes in the nematode life cycle, which can be used in the development of effective approaches for nematode control.

## 1. Introduction

*Meloidogyne enterolobii* is a species of pathogenic root-knot nematode that has attracted great attention and has been reported on worldwide in recent years [1,2,3]. This species is characterized by high infectivity and induces serious root knots on its host plant. It can even parasitize and propagate on cultivated resistant varieties carrying antigen genes, such as pepper (*N* gene), tomato (*Mi-1* gene) and cowpea (*Rk* gene), resulting in crop yield losses of more than 65% [4]. Therefore, it is considered to be one of the most destructive plant pathogenic nematodes. *M. enterolobii* was first discovered in 1983 from roots of the pacara earpod tree in Hainan Province, China [5]. In recent years, *M. enterolobii* has been reported in tropical and subtropical regions of Africa, Asia, North America, the Caribbean, and South America [6]. In China, *M. enterolobii* has been found in Guangdong, Guangxi, Fujian, and Hunan Provinces [7,8]. Field investigations showed that *M. enterolobii* was widely occurring and had replaced *M. incognita* as the dominant root-knot nematode species on fruits and vegetables in Hainan [9,10].

Collagens are a group of important structural proteins in nematodes, accounting for 80% of the components of nematode cuticle. Collagens can protect nematodes against adverse external environmental conditions, and play an important role in nematode morphogenesis, movement, and signal transduction [11]. In animal and human parasitic nematodes, collagen is also an important antigen for host immune system attack. Cuticle collagen is generally a small collagen with a molecular weight of about 30 kDa [12]. In *Caenorhabditis elegans*, there are 154 genes encoding the collagen protein family, which is involved in the formation of two different structures, the basement membrane and the cuticle. Genes encoding collagen have strict spatial and temporal expression patterns, as the expression levels are closely related to the life history and molting of the larvae, instead of being expressed in every stage of the life cycle. For example, the mRNA of *dpy-7* showed a peak of abundance approximately 4 h before each moult. By contrast, mRNA of *col-12* exhibited abundance peaks 4 h after each moult [13]. Studies have shown that single collagen mutations may cause significant morphological changes and severe loss of function in *C. elegans*, which manifest as reduced number of offspring; embryo or larval death; changes in epidermal morphology; changes in body shape, such as shortness and coarseness; and changes in locomotion morphology, such as body curl [14]. For example, RNA interference-type nematodes targeting the *C. elegans sqt-1* gene exhibited characteristics of short, thick body and germ cell variation [15]. The *dpy-2* and *dpy-10* genes of *C. elegans* are important in morphogenesis and other developmental events [16]. The *C. elegans dpy-5* gene encodes a cuticle procollagen, and *dpy-5*-deficient nematodes resulted in a short-body, dumpy phenotype, suggesting that *dpy-5* is required for normal cuticle formation [17].

There have been few reports on collagen in plant parasitic nematodes. Only a few collagen genes have been isolated from *M. incognita, M. javanica*, and *Globodera pallida*. The collagen gene *Lemmi* was first cloned from the cDNA sequence of *M. incognita*, which showed 63% similarity to *col-1* of *C. elegans*. It was confirmed that Lemmi protein was synthesized in the lower cortex of the stratum cuticle of female adults [18]. The collagen genes *gp-col-1* and *gp-col-2* were isolated from *G. pallida*, which were expressed only in female adult stage [19]. The collagen genes *Mj-col-3* and *Mj-col-5* of *M. javanica* were expressed in high abundance in the egg stage, but the transcription level decreased gradually in the parasitic stage [20]. The collagen gene *Mi-col-5*, isolated from *M. incognita*, was also highly expressed at the egg stage, indicating that this gene may function mainly at this stage [21]. Different developmental expression patterns of collagen genes in plant parasitic nematodes indicate different functional positions. However, the studies on collagen in plant parasitic nematodes are mainly confined to gene sequence cloning and expression-type analysis without specific function research. There is still no direct evidence for whether collagen is closely related to the movement, morphological maintenance, or other important reproductive developmental processes of plant parasitic nematodes.

Virus-induced gene silencing (VIGS) refers to the phenomenon that genes can be down-regulated or not expressed after the recombinant vector carrying the target gene fragment infects the plant by various methods. At present, this technology is widely used in tomato, tobacco, wheat, rice, barley, potato, and other crops [22,23,24,25]. VIGS plays an important role in plant functional genomics because of its advantages, such as simple technology, short research period, high infection efficiency and no need for genetic transformation [26]. In 1995, Kumagai et al. silenced the PDS gene for the first time in tobacco, and the plants showed bleaching symptoms in young leaves [27]. Since the first attempt to induce RNA interference (RNAi) of a target gene in plant-parasitic nematodes via the VIGS method, this technique has been widely used in the study of the gene function for plant-parasitic nematode interaction with the host plant [26]. *MiASB* silencing via VIGS resulted in significantly fewer galls on tomato seedlings [28]. The transcript of *Mi16D10* was knocked down via VIGS, resulting in a significant inhibition of nematode development. The up-regulation of the *Mi-vap-2* gene triggered by TRV-RNAi was transmitted to *M. incognita* progeny, which resulted in increased protein expression and promotion of pathogenicity [29]. 

To improve the knowledge of the role of collagen in *M. enterolobii*, we successfully isolated, cloned, and characterized a cuticle collagen gene, *Me-col-1*, from *M. enterolobii*. Further studies focused on the function of *Me-col-1* were conducted by RNAi in vitro or in vivo. The data obtained in this study revealed that Me-col-1 was crucial for productivity of *M. enterolobii*, as it may be involved in egg formation, further affecting the fecundity of *M. enterolobii*.

## 2. Materials and Methods

### 2.1. Nematodes and Plants

A *Meloidogyne enterolobii* population was isolated from the roots of the susceptible pepper in the field at Wenchang, Hainan. The single egg mass was inoculated on tomato (*Lycopersicon esculentum* cv. Super star) and cultured in a greenhouse. Eggs, preJ2s, parJ2s, J3/J4, and females were collected as previously described [30]. Seedlings of *Nicotiana benthamiana* were grown in a greenhouse at 25 °C (16 h light/8 h dark).

### 2.2. Cloning of Me-col-1 cDNA Sequence

Total RNA was extracted from *M. enterolobii* preJ2s by TRIzol (Invitrogen, Carlsbad, CA, USA) as described in the product specification. Both 3‘and 5’ RACE were carried out according to the instruction of GeneRacer^TM^ RACE kit (Invitrogen, Carlsbad, CA, USA). In detail, the first-strand cDNA with 3′ and 5′ linker (RACE template) was obtained by reverse transcription synthesis using the total RNA of preJ2s as templates. The specific primers for *Me-col-1* gene end amplification were designed according to the *M. enterolobii* transcriptome database built by our laboratory. The 5′ and 3′ end sequences of the *Me-col-1* gene were amplified using nested PCR combined with the RACE adaptor primers in kit. GeneRacer5′ primer and C-S1 were used in the first round of 5′ end sequence amplification, and GeneRacer5′ nested primer and C-S2 were used in the second round. GeneRacer3′ primer and M-A1 were used in the first round of 3′ end sequence amplification, and GeneRacer3′ nested primer and M-A2 were used in the second round. According to the obtained terminal sequences, full-length specific primers Col-F and Col-R were designed to amplify and verify the full sequence of *Me-col-1* cDNA. All primers used in this study are listed in Table 1.

### 2.3. Bioinformatic Analysis of Me-col-1 Sequence

The obtained fragment of Me-col-1 was sequenced by BGI Tech Solutions Co., Limited (Beijing Liuhe, Beijing, China). The open reading frame (ORF) was conducted on ORF Finder (http://www.ncbi.nlm.nih.gov/projects/gorf/gorf.html, accessed on 9 May 2020). The encoding protein domains were predicted on the InterPro website (https://www.ebi.ac.uk/interpro/, accessed on 10 October 2022). The protein molecular weight and isoelectric point were predicted using the online software PROTEIN MACHINE (http://us.expasy.org/tools/, accessed on 10 October 2022). The homology searches were conducted on BLASTP and BLASTX on the NCBI server (http://blast.ncbi.nlm.nih.gov/Blast.cgi, accessed on 10 October 2022). Then the homologous sequences were obtained from GenBank Database according to the following accession numbers: Mi-Col (AOG74800) from *M. incognita*, Mg-Col (KAF7629959) from *M. javanica*, Mj-Col (AAK83075) from *M. javanica*, Ab-Col (KAI6231065) from *Aphelenchoides besseyi*, Af-Col (KAI6242359) from *A. fujianensis*, Dd-Col (KAI1707436) from *Ditylenchus destructor*, Bx-Col (CAD5217526) from *Bursaphelenchus xylophilus*, and Ce-Col (AAA27991) from *C. elegans*. These sequences were aligned with Me-col-1 via CLUSTALW (https://www.genome.jp/tools-bin/clustalw, accessed on 10 October 2022) using default parameters. The phylogenetic tree was generated by MAGE and bootstrap values from 1000 replicates are shown at each node.

### 2.4. Developmental Expression Analysis

Total RNA was extracted separately from *M. enterolobii* at different life stages, as described above. A total of 350 ng of RNA were used for reverse transcription into cDNA using a PrimeScript™ RT reagent Kit with gDNA Eraser (Takara Bio Inc., Dalian, China). After five-fold dilution, 2 μL of cDNA were detected by reverse transcription-quantitative PCR (RT-qPCR) with primer pairs Col-QF/Col-QR and MeActF/MeActR (Table 1), respectively. The RT-qPCR assays were performed in the QuantStudioTM 6 Flex System (Applied Biosystems, Foster City, CA, USA) using TransStart^®^ Top Green qPCR SuperMix (Trans-Gen Biotech, Beijing, China). The transcript abundance of *Me-col-1* in each sample was normalized by the reference gene *Actin* (KF534787), and the relative transcript levels of target genes were calculated using the 2^–ΔΔCt^ method [31]. Three independent biological replicates were conducted while each RT-qPCR amplification was run in triplicate.

### 2.5. Heterologous Expression of Protein Me-col-1

Using the first strand cDNA as templates, the ORF region of the *Me-col-1* gene was amplified with the primers Col-BamHI and Col-NotI (Table 1). Then *Me-col-1* fragments and the prokaryotic expression vectors pET-32a (+) were digested by *BamH*I and *Not*I restriction enzymes individually. The digestion products were ligated together to obtain recombinant plasmids of pET-32a-COL. The recombinant plasmids pET-32a-COL were transformed into *Escherichia coli* BL21 (DE3) competent cells by heat shock, and the positive transformants were selected for the following protein expression. The recombinant *E. coli* strain containing pET-32a-COL or pET-32a (+) vector was cultured in LB media supplemented with 50 μg/mL ampicillin at 37 °C by shaking until the OD600 value reached 0.6. The protein expression of *Me-col-1* was induced with isopropyl β-_D_-1-thiogalactopyranoside (IPTG) with the final concentrations of 0.2 mM, 0.4 Mm, 0.8 mM, and 1.0 mM, followed by incubation at 30 °C for 12 h. Finally, the cells were collected by centrifugation and lysed by sonication to obtain the total proteins. The expression of the recombinant *Me-col-1* protein was detected using 12% SDS polyacrylamide gel electrophoresis (SDS-PAGE) and Coomassie brilliant blue R-20 staining. In this experiment, IPTG-induced cells containing pET-32a (+) vector were used as negative controls.

### 2.6. dsRNA Preparation and In Vitro RNAi of Me-col-1

The *Me-col-1* dsRNA was synthesized and purified with a MEGAscript RNAi Kit (Thermo Fisher Scientific, Waltham, MA, USA) with the primers Col-IF and Col-IR (Table 1), according to the manufacturer’s instructions. Then, in vitro RNAi was conducted by soaking, as previously described [32]. First, approximately 500 freshly hatched preJ2s of *M. enterolobii* were soaked in 200 μL of PBS buffer containing 2 mg/mL *Me-col-1* dsRNA, 0.5% resorcinol, 0.05% gelatin, and 3 mM spermidine. Nematodes soaked in water or buffer without dsRNA were used as controls. The soaking time was set to 12 h, 24 h, and 48 h in a dark at room temperature on a rotator. At the end of soaking, the nematodes were washed repeatedly with nuclease-free water and recovered in water for 2 h. Finally, the mobility of the nematodes was examined under a microscope and the number of dead nematodes were counted in each treatment. The experiment was performed with three independent replicates.

### 2.7. In Planta RNAi

The specific fragment of the *Me-col-1* gene was amplified with primers DcolF and DcolR (Table 1), and was cloned into the tobacco rattle virus vector pTRV2-EX to construct a pTRV2-Col-silencing recombinant plasmid. The plasmids were transformed into Agrobacterium tumefaciens strain GV3101. The expression of TRV was obtained by mixing pTRV2-Col and pTRV1 at a 1:1 ratio and inoculating 2–3 leaves of *N. benthamiana* at 4 weeks old. Control pTRV2-EX was inoculated in the same way. After 7 days of inoculation, total RNA was extracted from the new root tissue of *N. benthamiana* and reverse transcribed. The expressions of gene-encoding TRV coat protein in the roots were verified by PCR amplified with primers TRVF and TRVR (Table 1). Then, 2000 freshly hatched preJ2s of *M. enterolobii* were inoculated with 5 plants of *N. benthamiana* in each treatment. The number of root knots, eggs, and hatched preJ2s were counted after inoculation for 35 days. The ratio of the final PreJ2 population density (Pf) to the initial PreJ2 population density (Pi) was defined as multiplication rates. The experiment was performed with three independent replicates.

### 2.8. Statistical Analysis

In this study, statistical analysis was performed using OriginPro 2021 software (OriginLab, Northampton, MA, USA). All values were expressed as mean ± standard error. One-way ANOVA tests were used to analyse significant differences between treatments by Tukey’s Test, and the *p* value was set as 0.01.

## 3. Results

### 3.1. Sequence Characterization and Analysis of Me-col-1 Gene

According to the transcriptome data obtained from the preJ2s, the 5′ and 3′ end sequences of *Me-col-1* cDNA were cloned by RACE-PCR. The full length of *Me-col-1* cDNA is 1211 bp, containing a 71 bp of 5′ untranslated regions (UTRs), 93 bp of 3′ UTRs, and 1047 bp of complete open reading frame. The 3′ UTRs contains the poly(A) tail of 20 bp. The *Me-col-1* sequence has been submitted under GenBank accession number of KU350654.

**Figure 1:**
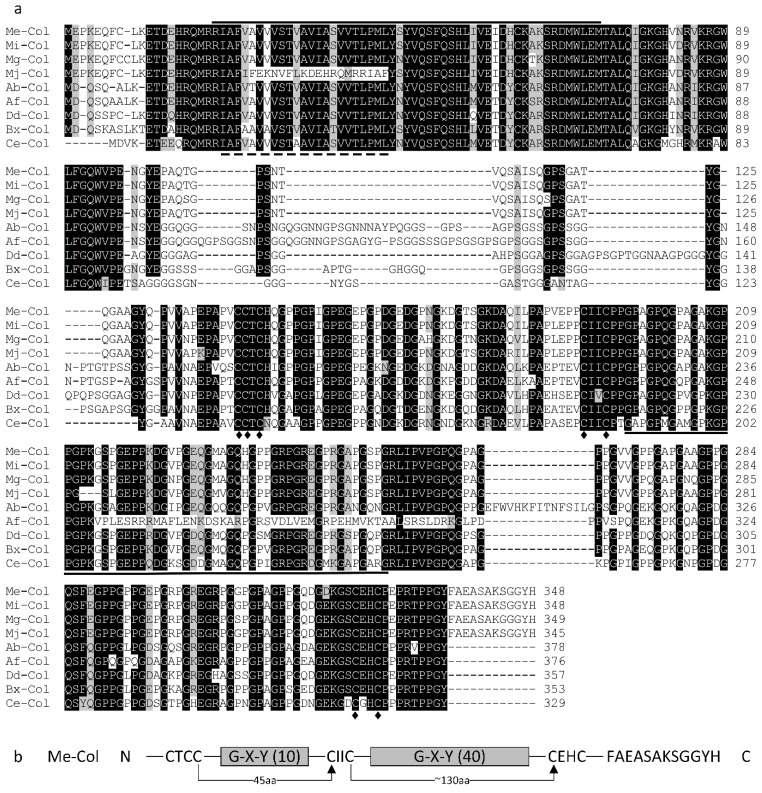
Sequence characterization of *Me-col-1* gene. (**a**) Multiple sequence alignment of Me-col-1 with collagens of other nematodes. Shading characters indicate identical (black) or similar (grey) amino acids. The transmembrane region sequence of all Cols is denoted with dotted lines. Nematode cuticle collagen N-terminal domain is marked with overbar. The conserved collagen triple helix repeat domain is underlined. Conserved cysteine residues are marked with rhombuses. (**b**) Conserved pattern of Me-col-1 cysteine residues. The numbers in parentheses represent the number of Gly-X-Y repeats.

The *Me-col-1* protein (GenBank accession number ANH56393) consists of 348 amino acid residues, with a predicted molecular weight of 35.09 kDa and an isoelectric point (pI) of 6.30. *Me-Col-1* contains the nematode cuticle collagen N-terminal domain (20–72 aa), the conserved collagen triple helix repeats domain (196–253 aa), and 50 Gly-X-Y repeats (Figure 1a). In addition, the *Me-Col-1* protein contains one transmembrane domain (21–43 aa) and no signal peptide was found, indicating that Me-Col-1 belongs to the membrane protein family.

Alignment of the deduced amino acid sequences showed high homology identities of Me-col-1 to cuticle collagens (cols) of other nematodes. The Me-col-1 protein sequence showed 99.14% identity with Mi-Col, 94.56% identity with Mg-Col, 91.38% identity with Mj-Col, 61.48% identity with Ab-Col, 53.42% identity with Af-Col, 68.65% identity with Dd-Col, 69.04% identity with Bx-Col, and 62.75% identity with Ce-Col (Figure 1a).

**Figure 2:**
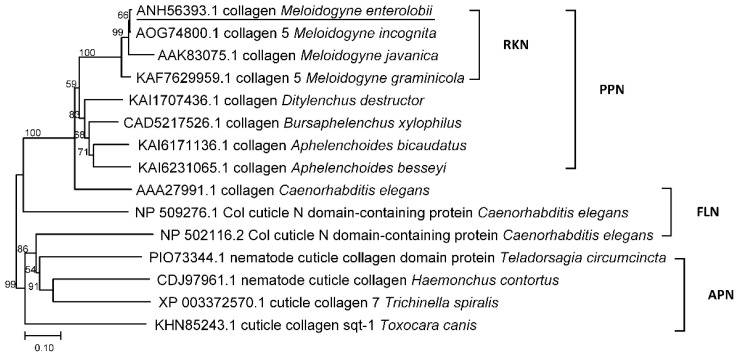
Phylogenetic analysis of Me-col-1 (underline) with collagens of other nematodes. RKN, root-knot nematode; PPN, plant-parasitic nematode; FLN, free-living nematode; APN, animal-parasitic nematode.

Based on the classification and the pattern of conserved cysteine residues [13], *Me-col-1* belongs to group 2 of the cuticle collagen gene, and the carboxy terminus of Me-col-1 is also 12 amino acids longer than the other members of group 2 and has an additional tyrosine residue (Figure 1b). The phylogenetic tree involving the collagens was isolated from root-knot nematodes, plant-parasitic nematodes, free-living nematodes, and animal- parasitic nematodes. Me-col-1 and all collagens from root-knot nematodes were included in a clade (Figure 2). 

### 3.2. Prokaryotic Expression of Me-col-1 Protein

**Figure 3:**
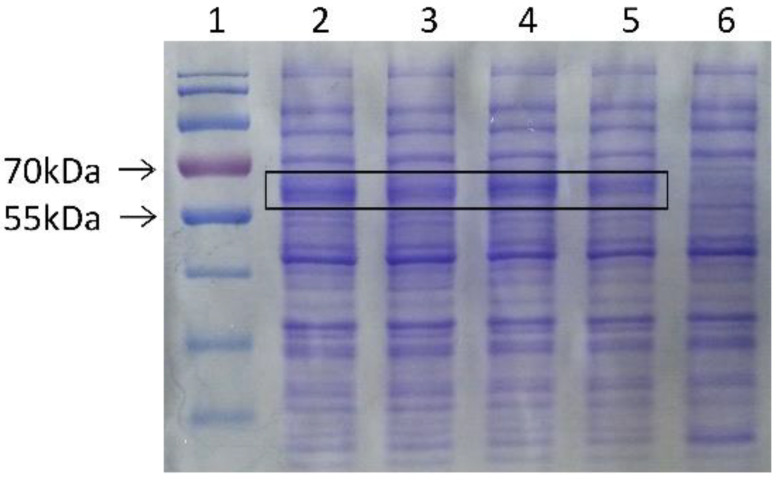
SDS-PAGE analysis of Me-col-1 fusion protein. Lane 1, marker; Lane 2–5, Me-col-1 fusion protein induced by IPTG at final concentrations of 0.2 mM, 0.4 Mm, 0.8 mM, 1.0 mM, respectively; Lane 6, pET-32a.

The recombinant expression vector pET-32a-Col was constructed and transferred into *E. coli* strain BL21 (DE3) for prokaryotic expression. The results of SDS-PAGE showed that the Me-col-1 fusion protein was successfully induced by IPTG at concentrations of 0.2 mM, 0.4 Mm, 0.8 mM, and 1.0 mM, and the specific fragment with the size of about 61 kDa was obtained. After removing the size of tag sequence (26 kDa) in the vector, the remaining protein size was consistent with the predicted size of Me-col-1 (35 kDa). No Me-Col-1 fusion protein was found in extracted proteins from cells containing pET-32a (Figure 3).

### 3.3. Temporal Expression Assays

**Figure 4:**
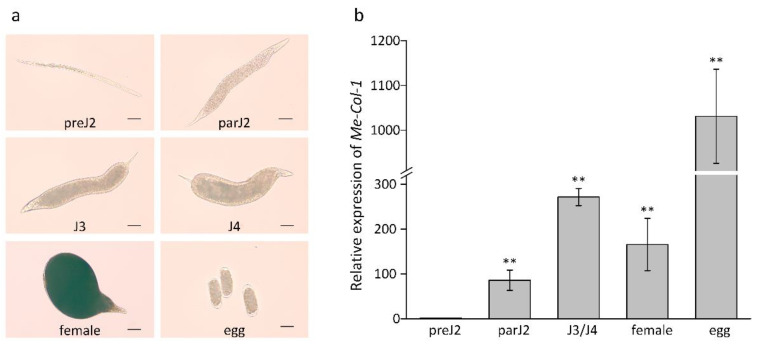
(**a**) The characterization of nematodes at preJ2, parJ2, J3, J4, female, and egg life stages. The length of the ruler is 20 um; (**b**) Expression level analysis of Me-col-1 at different developmental stages of *M. enterolobii*, including preJ2, parJ2, J3/J4, female, and egg life stages. Each bar represents the mean of RT-qPCR reactions run in triplicate, with use of standard error. Significant differences between values were derived by Tukey’s Test (** *p* ≤ 0.01). Three independent experiments were performed with similar results.

To evaluate *Me-col-1* gene expression during *M. enterolobii* developmental stages, the transcription of *Me-col-1* was quantified using RT-qPCR at 5 different developmental stages of *M. enterolobii* (Figure 4a). The *Actin* gene of *M. enterolobii* was used as endogenous control and the transcript level of *Me-col-1* in preJ2 was used as a reference. The expression of *Me-col-1* was the lowest in preJ2, and then increased by 85.7-fold in parJ2, 271.5-fold in J3/J4, and 165.5-fold in females, respectively. Notably *Me-col-1* transcription peaked in the eggs, in which the relative expression level increased dramatically by 1031.1-fold when compared with that in preJ2 (Figure 4b). These results indicated that *Me-col-1* is most highly expressed in the parasitic and egg stages of *M. enterolobii*. It is speculated that *Me-col-1* is involved in nematode feeding and egg formation.

### 3.4. Me-col-1 In Vitro RNAi

**Figure 5:**
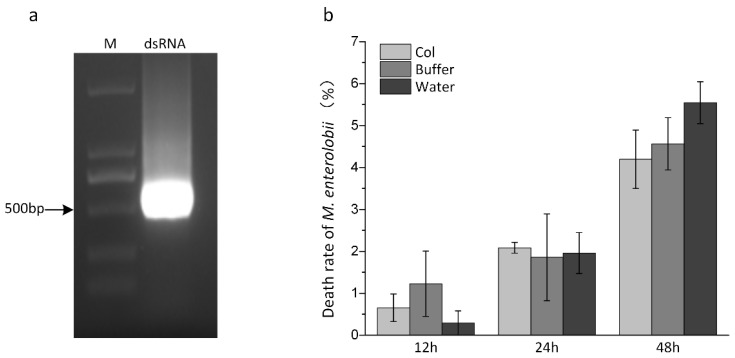
(**a**) Electrophoretic results of Me-col-1 dsRNA after purification and recovery. M, marker; (**b**) Death rate of *M. enterolobii* after in vitro RNAi.

To test the function of Me-col-1 in *M. enterolobii*, we synthesized the *Me-col-1* (444 bp) dsRNA fragment by introducing the T7 promoter sequence, and then detected the dsRNA concentration of 4233 ng/ul with a spectrophotometer (Figure 5a). The statistical results showed that there was no significant difference in the death rate among different treatments, and the death rate of larvae within 48 h was within 5% (Figure 5b). The experiment failed to effectively induce the down-regulation of *Me-col-1* gene expression, which was speculated to be due to the low transcript abundance of *Me-col-1* gene in preJ2, and the failure of dsRNA to effectively enter the expressing cells by the feeding method, leading to the off-target effect.

### 3.5. Me-col-1 In Vivo RNAi

**Figure 6:**
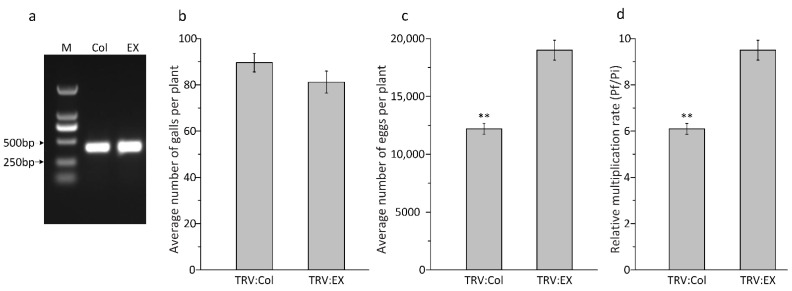
Effect of in planta RNAi of Me-col-1. (**a**) The TRV cp gene in *N. benthamiana* roots was detected by PCR 7 days after TRV inoculation. M: marker, Col: TRV2-Col, EX: empty vector control TRV2-EX; (**b**) the numbers of galls in the *N. benthamiana* roots; (**c**) average number of eggs per plant; (**d**) the multiplication rate (Pf/Pi) of TRV inoculated plants. Data are presented as means from 10 plants. The independent experiments were repeated twice with similar results. (** *p* ≤ 0.01).

To test the function of *Me-col-1* in the parasitism of *M. enterolobii*, we introduced the VIGS system to silence *Me-col-1* in *vivo*. After TRV inoculation for 7 days, fragments of the TRV coat protein gene (447 bp) were detected by PCR in the roots of *N. benthamiana* inoculated by TRV2-Col or empty vector (Figure 6a). After being inoculated for 35 days, root knots were observed in all treatments, and the mean disease indexes of TRV2-Col treatment and empty vector control were 40.7% and 42.3%, respectively, without significant difference (*p* ≤ 0.05, Figure 6b). On average, there were about 6800 fewer eggs per plant in TRV2-Col treatment than in the empty vector control (Figure 6c). The multiplication rates (Pf/Pi) of TRV2-Col treatment and empty vector control were 6.1 and 9.5, respectively (Figure 6d). The reproductive rate of TRV2-Col was significantly lower than that of the empty vector control (*p* ≤ 0.01), indicating that the *Me-col-1* gene plays an important role in the reproductive process of the nematode, which is speculated to be closely related to reproductive development.

## 4. Discussion

The nematode cuticle is an ordered, multi-layered, extracellular structure formed by macromolecules secreted by the upper cuticle cells. As the outer skeleton of the nematode, the cuticle can maintain its normal morphology and integrity and play an important role in many physiological functions of the nematode, such as movement, growth and development, osmotic regulation, and so on. It also provides a permanent protective barrier for the nematode to defend against pathogen invasion and environmental stress. Collagen is a major component of the nematode cuticle [33]. During the growth and development of the nematode, new cuticle is constantly synthesized through the molting process to ensure normal growth and provide the necessary protective barrier [34]. When the collagen gene in the synthesis pathway is mutated, the formation of the cuticle will be defective, which can lead to morphological variations such as short body, floating cuticle, and distorted body, and also cause embryonic and larval death [13].

In this study, a new collagen gene, *Me-col-1*, was successfully cloned from *M. enterolobii* genome. Bioinformatics analysis showed that the encoded protein contained a transmembrane N-terminal region and had no signal peptide sequence, indicating that it is a typical transmembrane protein. The sequence alignment of *Me-col-1* and the phylogenetic analysis of Me-col-1 with other collagens in nematodes showed that all nematode collagens were more closely related to plant parasitic nematodes than to collagens from free living nematodes and animal parasitic nematodes, which proved that this type of protein is conserved in plant parasitic nematodes.

Me-col-1 has the same conserved cysteine residue pattern as Mi-col-5, Mj-col-5 [20,21]. The carboxy terminus of Me-col-1 is 12 amino acids longer than the other members of group 2 with an additional tyrosine residue. Because cuticle collagens are cross-linked by nonreducible covalent bonds that involve tyrosine residues [35]. The extra tyrosine residues in *Me-col-1* suggests a different cross-linking pattern compared with the other members of group 2.

RT-PCR was used to detect the relative expression level of *Me-col-1* in different developmental stages of *M. enterolobii*. The results showed that *Me-col-1* was expressed in all stages, and the expression level of *Me-col-1* in eggs was significantly higher than that in other stages, and the expression level of preJ2 was the lowest. This result is similar to that of *Mi-col-5* and *Mj-col-5* [20,21]. The product of *Mj-col-5* is not a component of eggshell. Given that *Me-col-1* shares 91.38% homology with *Mj-col-5*, it is speculated that *Me-col-1* is not a component of eggshell, either. The high expression in eggs may be related to the formation of the embryonic cuticle and may also be involved in the molting of larval instars, and at least part of the Me-col-1 transcript detected in females may be due to unlaid eggs.

Mutation of the *emb-9* gene, the α1 (IV) collagen gene of *C. elegans*, resulted in temperature-sensitive lethality in late embryonic development [36]. Most of the mutations in type IV genes cause embryo lethality, suggesting that embryogenesis requires a normal basement membrane [14]. Strong loss-of-function mutations in *sqt-3*-encoding COL-1 collagen result in embryonic lethality and early larval arrest, characterized by the same retracted Dpy phenotype as in *dpy-31* mutants [37]. Loss-of-function mutations in *dpy-31* lead to cuticle defects, abnormal morphology, and embryonic lethality, suggesting that *dpy-31* is essential for collagen exoskeleton formation. However, the RNAi of *dpy-31* occasionally leads to embryonically lethal phenotypes in progeny, similar to those of *dpy-31* mutants. The *dpy-31* gene is relatively insensitive to RNAi [38]. In our study, *Me-col-1* in vitro RNAi failed to cause lethality of preJ2s, indicating a different effect for nematode survival. However, this conclusion needs to be further verified in future studies. As shown in RT-qPCR data, the transcript level of *Me-col-1* was quite low at the preJ2 stage, so silencing of *Me-col-1* probably has little impact on preJ2s of *M. enterolobii*. On the contrary, no lethality to preJ2s could result from the off-target effect of RNAi, as dsRNA failed to effectively enter the expressing cells.

Furthermore, in vivo RNAi techniques were used to analyze the effect of *Me-col-1* silencing. *Me-col-1* expression in tobacco roots was induced by VIGS. *Me-col-1* silencing is caused by nematodes feeding on target fragments. Although there was no significant difference in average disease index between the TRV2-Col treatment and the empty vector control, the Pf/Pi value of TRV2-Col treatment was significantly lower than that of the empty vector control. This indicated that *Me-col-1* plays an important role in the reproduction of the root knot nematode. This study basically clarified the expression pattern and functional status of *Me-col-1* in root knot nematodes, which lays a theoretical foundation for screening the target genes of RNAi resistance engineering and developing a new green strategy for effective control of root knot nematode diseases.

## Figures and Tables

**Table 1:** List of primers used in this study.

Primer Name	Primer Sequences (5′–3′)
GeneRacer5′ primer	CGACTGGAGCACGAGGACACTGA
C-S1	CCCTGTCCATAGGTTGCCCCAC
GeneRacer5′ nested primer	GGACACTGACATGGACTGAAGGAGTA
C-S2	TCCAGGCACGGGAATGAGACGA
GeneRacer3′ primer	TACCGTCGTTCCACTAGTGATTT
M-A1	GGGGCAACCTATGGACAGGGAGC
GeneRacer3′ nested primer	CGCGGATCCTCCACTAGTGATTTCACTATAGG
M-A2	CTGGTCGTCTCATTCCCGTGCCT
Col-F	ATGGAACCTAAAGAGCAG
Col-R	TTAATGATATCCACCACTTTTTGCACTTGC
Col-QF	GCTCTCCTGGTCGTCTCATT
Col-QR	ACAACTGCCCTTATCTCCG
MeActF	ACGGTCAAGTCATTACTGTTGGAAA
MeActR	GTAAAGGTCTTTACGGATGTCAATG
Col-BamHI	CGGATCCGAACCTAAAGAGCAGTTTTGC
Col-NotI	TTGCGGCCGCAATGATATCCACCACTTTTTGCACT
Col-IF ^1^	TAATACGACTCACTATAGGGAGATGTAGCGGTTGTTGTTTCG
Col-IR ^1^	TAATACGACTCACTATAGGGAGATTTCCATTAGGTCCATCCTC
TRVF	CTGGGTTACTAGCGGCACTGAATA
TRVR	TCCACCAAACTTAATCCCGAATAC
DcolF	TGTAGCGGTTGTTGTTTCG
DcolR	TTTCCATTAGGTCCATCCTC

^1^ T7 polymerase promoter sequences are underlined.

## Data Availability

Not applicable.
